# Genetic Drift Shapes the Evolution of a Highly Dynamic Metapopulation

**DOI:** 10.1093/molbev/msac264

**Published:** 2022-12-06

**Authors:** Pascal Angst, Camille Ameline, Christoph R Haag, Frida Ben-Ami, Dieter Ebert, Peter D Fields

**Affiliations:** Department of Environmental Sciences, Zoology, University of Basel, Basel 4051, Switzerland; Department of Environmental Sciences, Zoology, University of Basel, Basel 4051, Switzerland; Evolutionary Biology, Instituto Gulbenkian de Ciência, Oeiras 2780-156, Portugal; CEFE, Université de Montpellier, CNRS, EPHE, IRD, Montpellier 34293, France; Tvärminne Zoological Station, University of Helsinki, Hanko 10900, Finland; Tvärminne Zoological Station, University of Helsinki, Hanko 10900, Finland; George S. Wise Faculty of Life Sciences, School of Zoology, Tel Aviv University, Tel Aviv 69978, Israel; Department of Environmental Sciences, Zoology, University of Basel, Basel 4051, Switzerland; Tvärminne Zoological Station, University of Helsinki, Hanko 10900, Finland; Department of Environmental Sciences, Zoology, University of Basel, Basel 4051, Switzerland; Tvärminne Zoological Station, University of Helsinki, Hanko 10900, Finland

**Keywords:** metapopulation, genomics, turnover dynamics, daphnia, cladocera, crustacea

## Abstract

The dynamics of extinction and (re)colonization in habitat patches are characterizing features of dynamic metapopulations, causing them to evolve differently than large, stable populations. The propagule model, which assumes genetic bottlenecks during colonization, posits that newly founded subpopulations have low genetic diversity and are genetically highly differentiated from each other. Immigration may then increase diversity and decrease differentiation between subpopulations. Thus, older and/or less isolated subpopulations are expected to have higher genetic diversity and less genetic differentiation. We tested this theory using whole-genome pool-sequencing to characterize nucleotide diversity and differentiation in 60 subpopulations of a natural metapopulation of the cyclical parthenogen *Daphnia magna*. For comparison, we characterized diversity in a single, large, and stable *D. magna* population. We found reduced (synonymous) genomic diversity, a proxy for effective population size, weak purifying selection, and low rates of adaptive evolution in the metapopulation compared with the large, stable population. These differences suggest that genetic bottlenecks during colonization reduce effective population sizes, which leads to strong genetic drift and reduced selection efficacy in the metapopulation. Consistent with the propagule model, we found lower diversity and increased differentiation in younger and also in more isolated subpopulations. Our study sheds light on the genomic consequences of extinction–(re)colonization dynamics to an unprecedented degree, giving strong support for the propagule model. We demonstrate that the metapopulation evolves differently from a large, stable population and that evolution is largely driven by genetic drift.

## Introduction

Metapopulations, i.e., subpopulations interconnected by extinction–(re)colonization dynamics, are ubiquitous (Hanski et al. 2017). They differ from classical, large and stable, populations (with or without spatial structure) due to this extinction–(re)colonization dynamic, a feature that introduces recurrent genetic bottlenecks during the founding of new subpopulations (Hanski 1999; Hanski et al. 2017; Wang and Altermatt 2019). In early population genetic models, extinction–(re)colonization dynamics received little attention. Only after Levins (1969) first introduced the concept of a metapopulation, primarily to address ecological questions, population geneticists sought to discover how metapopulation dynamics affect variation in genetic diversity and differentiation (Giles and Goudet 1997; McCauley 1989; McCauley et al. 1995; Slatkin 1977; Wade and McCauley 1988; Whitlock 1992; Whitlock and McCauley 1990). As suggested by the propagule model (Slatkin 1977), new subpopulations are founded when empty habitat patches are colonized by one or a few individuals, often originating from a single source population. The genetic bottlenecks can then lead to high genetic differentiation among new subpopulations and low diversity within subpopulations, and thus, low effective population sizes. These effects, in turn, lead to increased genetic drift and genetic load (Cosentino et al. 2012; Montero-Pau et al. 2018; Saastamoinen et al. 2018; Tortajada et al. 2009; Waples 2017). Conversely, mutation and gene flow can have the opposite effect in metapopulations (Hanski and Gaggiotti 2004; Pannell and Charlesworth 2000): with a continued occurrence of mutations and influx of immigrants, older subpopulations might become genetically more diverse. Due to the exchange of migrants, subpopulations may become less differentiated from each other than newly founded subpopulations.

Ecological factors that contribute to evolution may vary among metapopulations (Bonte and Bafort 2019; Hedrick and Gilpin 1997; Ingvarsson et al. 1997; Molofsky and Ferdy 2005; Pannell and Charlesworth 2000), with turnover (extinction–(re)colonization dynamics) and population size (often correlated with habitat size) playing an important role. These factors affect the probability of allele fixation in (sub)populations (Charlesworth 2009; Johri et al. 2021; Whitlock 2003). Specifically, the fixation probability of an emerging allele depends not only on its selection coefficient, but also on the effective size, *N_e_*, of subpopulations and the degree of population structure (Charlesworth et al. 2003; Vuilleumier et al. 2008; Whitlock 2003). Variations in population size and structure, as well as their influence on the evolutionary process, can be studied using population genetic summary statistics, such as (non)synonymous genomic diversity, π_N_ and π_S_, and the rate of (non)adaptive nonsynonymous substitutions, *ω_NA_* and *ω_A_*. Thus, population genetics can help to determine the ecological setting of a particular metapopulation and give insight into how evolutionary mechanisms differ among metapopulations (Gaggiotti and Foll 2010).

Hedrick and Gilpin (1997) were among the first to discuss the factors that influence evolution in metapopulations, but these issues have not been much addressed in metapopulation genetic studies (but see, e.g., Montero-Pau et al. (2018) for a theoretical model). Metapopulations with stable subpopulations resemble the island model of connected Wright–Fisher populations (Wright 1931). In these metapopulations, genetic bottlenecks are rare or weak, so evolution is expected to be predominantly driven by natural selection (Ronce 2007; Whitlock 2004), and local adaptation can help to maintain or promote population differentiation and counteract gene flow that would otherwise reduce subpopulation's differentiation (Szép et al. 2021). The benthic reef gastropod *Haliotis laevigata* in southern Australia is an example of this kind of metapopulation with large effective population sizes, high connectivity, and low turnover (Sandoval-Castillo et al. 2018).

Strong and frequent bottlenecks can lead to small effective population sizes in which genetic drift predominates (Charlesworth et al. 2003). This process, in turn, weakens natural selection against deleterious mutations and rates of adaptive evolution. The North American Gila Trout (*Oncorhynchus gilae*) shows this pattern of metapopulation with small effective population sizes, low gene flow, and genomic bottlenecks (Camak et al. 2021). The bottlenecks and the associated low effective population sizes accelerate the accumulation and fixation of deleterious mutations, which reduces the mean fitness, referred to as local drift load (Whitlock 2004). In extreme cases, it results in a mutational meltdown of populations (Lynch et al. 1995). Gene flow can counteract this process, introducing new genotypes into a population. In case of hybrid offspring between immigrants and local residents, high drift load can lead to a fitness advantage via hybrid vigor and the subsequent purging of deleterious mutations (Ebert et al. 2002; Whitlock et al. 2000).

Systems with clearly defined subpopulations facilitate the study of metapopulation dynamics and their effect on the evolutionary process. Pond-dwelling organisms occur in distinct water bodies, making population boundaries easy to define. Here, we focus on a pond-dwelling species, the cyclically parthenogenetic microcrustacean *Daphnia magna*, which forms a large metapopulation on the Skerry Islands of southwestern Finland and along the Swedish east coast. As previous findings have suggested, this metapopulation follows the propagule model and is highly dynamic, i.e., characterized by small and unstable subpopulations, high extinction–(re)colonization dynamics, and strong colonization bottlenecks (Altermatt and Ebert 2010; Ebert et al. 2013; Fields et al. 2018; Zumbrunn 2011). A long-term survey of this metapopulation (Dubart et al. 2020; Ebert et al. 2013; Pajunen and Pajunen 2003) has revealed high turnover rates: of the 20% of the rock pools that contain *D. magna* subpopulations, about 20% go extinct every year, and about 5% of the empty ponds are colonized per year. This metapopulation has been shown to be an “inverse mainland-island” type of metapopulation (Altermatt and Ebert 2010), where the pool of migrants primarily comes from small ponds with small subpopulations. Such small ponds dry up more likely exposing the sediment-borne resting stages of *D. magna* to wind and animals. Empty habitat patches are primarily colonized (∼90% of the time) by single colonizers that then undergo clonal expansion (Haag et al. 2005). Isolated aspects of the system are well understood using phenotypic data (Lohr and Haag 2015) and genetic marker analysis (Haag et al. 2005; Walser and Haag 2012). For example, bottlenecks lead to low genetic diversity and genetic load (Haag et al. 2005), while immigration and subsequent hybridization lead to selection for hybrid genotypes (hybrid vigor) and elevated effective migration rates (Ebert et al. 2002). However, because multiple of these aspects act together and occur at different frequencies in time and space, it raises the question of the contribution of genetic drift and natural selection to the evolution in this metapopulation. For example, how does evolution at (non)synonymous sites in the nuclear genome vary in this metapopulation compared with in large stable populations?

In this *D. magna* metapopulation genomic study, we use allele frequency and ecological data to test our hypotheses about (1) genomic diversity, (2) population differentiation, and (3) (non)adaptive evolution. Our objective is to understand how this metapopulation evolves and, more generally, how evolution in metapopulations differs from the evolution in larger, stable populations. Considering Haag et al. (2005), who partially explained genetic diversity by a pond's age and its distance from the sea based on three allozyme markers in the same metapopulation and suggested that populations in less stable ponds closer to the sea face a higher risk of extinction, we revisit the effects of age, ecology, and geography on genomic diversity using whole-genomic data. We expect source sampling under the propagule model in our system to lead to lower genomic diversity in newly established *D. magna* subpopulations than in older ones. Regarding population differentiation, recurrent genomic bottlenecks might lead to high differentiation between founder populations, which might, however, erode over time due to gene flow (Haag et al. 2005). This high differentiation between founder populations could prevent a pattern of genetic isolation-by-distance (IBD), which is seen in larger scale data for *D. magna* (Fields et al. 2015), across small geographic distances. We further investigate the relative strength of natural selection versus genetic drift using statistics that estimate the proportion of (non)synonymous polymorphisms like π_N_ and π_S_ and (non)adaptive substitutions like *ω_NA_* and *ω_A_*. Because of recurring bottlenecks, *N_e_* should be low, which might weaken the efficiency of natural selection and strengthen the effect of genetic drift. Under this scenario, we predict a genomic signature of weak purifying selection, i.e., an excess of nonsynonymous polymorphisms (high ratio of π_N_/π_S_), and few polymorphisms fixed by adaptation (low *ω_A_*). By understanding the variation in genomic diversity, genomic differentiation, and (non)adaptive evolution in this metapopulation, we try to unravel the general principles of evolution in metapopulations and how they evolve differently from large, stable, panmictic populations using empirical and simulated data.

## Materials and Methods

### Study System

This study uses samples from a natural *Daphnia magna* metapopulation located on the Tvaerminne archipelago in southwestern Finland (59°50′ N, 23°15′ E). *Daphnia magna* is a Holarctic-distributed, freshwater planktonic crustacean that inhabits small rock pools on the islands of this archipelago. The rock pools (mean volume about 300 l) are depressions in the bare rock of the islands that fill with rainwater but also sometimes collect seawater. We call them rock pools or ponds to avoid confusing them with “pools” from our genomic pool-seq samples. The shallow rock pools are mostly frozen solid in winter; many dry up during the summer as well. Environmental variables including pond geometry, water salinity, humic acid content, pH, calcium concentration, distance to the sea, and height above sea level are available for all ponds (Ebert et al. 2013; Pajunen and Pajunen 2003; Ranta 1979). Since 1982, these ponds have been surveyed biannually for the presence of *D. magna*, and since 2007, we have assayed *D. magna* samples from these ponds for parasitic infections (D. Ebert, unpublished data). *Daphnia magna* is a cyclic parthenogen; sexual reproduction results in resting eggs that allow it to survive the winter freezes and the drying out of ponds during the summer. These resting eggs disperse passively by wind, water, or birds and are, therefore, crucial for the migration and colonization of vacant habitat patches. *Daphnia magna* also reproduces asexually, which allows clonal expansion after colonization.

### Samples and Sequencing

**Fig. 1.**
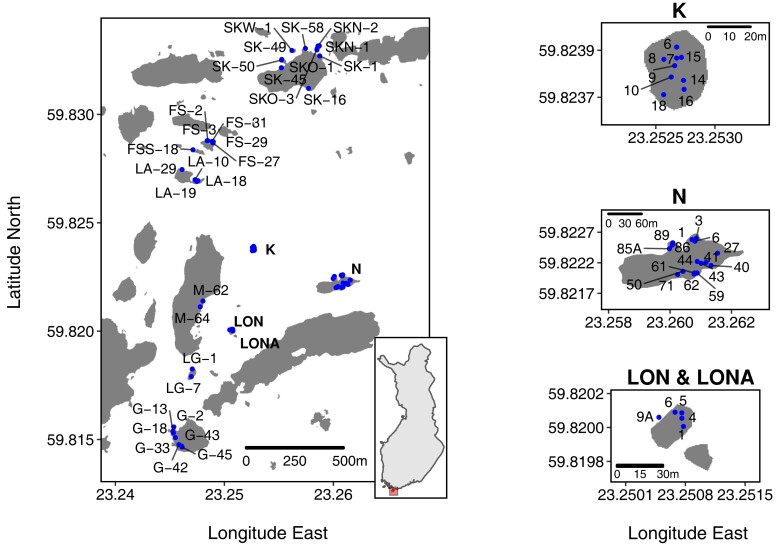
Geographic locations of sampled ponds (subpopulations). Large map shows the overview of the study site, with a small inset map of Finland indicating the location of the study site (small square in Southern Finland). Islands K, N, and LON/LONA are enlarged in separate maps to improve spatial resolution. Each pond has a unique identifier composed of its island's ID (see table 1) and a consecutive number. In the study area are about nine times more habitat patches than shown here. These other habitat patches did not have *D. magna* populations at the time of sampling.

Our aim was to collect *D. magna* from all occupied ponds (subpopulations) in the core sampling area in late May/early June of 2014 (fig. 1 and table 1, supplementary table S1, Supplementary Material online) in order to sequence the pooled genotypes, called pool-seq. Pool-seq is a powerful and cost-efficient way to estimate the genome-wide allele frequencies of populations, as it provides allele frequency estimates of SNPs that are mostly comparable to individual-based sequencing at less cost and with less time (Chen et al. 2022; Dorant et al. 2019; Gautier et al. 2013; Kurland et al. 2019). We collected random subpopulation samples by sieving with hand-held plankton nets through the ponds, aiming for 50 animals per pond and excluding ponds with very small populations at the time of sampling to avoid disrupting natural dynamics. In total, we collected 62 subpopulation samples from 13 islands (fig. 1 and table 1). Collected animals started a 3-day regime of antibiotics within 24 h of collection (Fields et al. 2018) and were fed dextran beads (Sephadex “Small” by Sigma Aldrich: 50 μm diameter) at a concentration of 0.5 g/100 ml to evacuate their gut content and reduce nontarget DNA sequencing. Whole animals were then stored in RNAlater (Ambion) and kept at minus 20°C, until we extracted DNA.

**Table 1.** Island Information.

Island	Island	Latitude/Longitude	*N*
Nameless skerry south of Fyrgrundet	FS	59.8286/23.2485	5
Nameless skerry more south of Fyrgrundet	FSS	59.8283/23.2476	1
Granbusken	G	59.8152/23.2467	7(−1)
Flatgrund	K	59.8238/23.2527	9
Lasarettet	LA	59.8273/23.2461	4
Nameless skerry	LG	59.8180/23.2470	2
Nameless skerry	LON	59.8200/23.2506	5
Melanskar	M	59.8215/23.2470	2
Storgrundet	N	59.8221/23.2593	16
Skallotholmen	SK	59.8319/23.2572	6(−1)
Nameless skerry north of Skallotholmen	SKN	59.8332/23.2587	2
Nameless skerry east of Skallotholmen	SKO	59.8330/23.2584	2
Nameless skerry west of Skallotholmen	SKW	59.8329/23.2563	1

Latitude and longitude data are obtained from Google maps. *N* , Number of sampled ponds (subpopulations); the two samples where the sequencing failed are shown as “−1”.

For DNA extraction, samples were thawed, the RNAlater was removed, and the samples were washed twice with water. We added 500 μl extraction buffer (Qiagen GenePure DNA Isolation Kit) to the sample tube and ground the sample using a plastic pestle. Then, we added 20 μl Proteinase K for overnight incubation at 55°C, after which we added 20 μl RNAse for RNA digestion for one hour at 37°C. For protein removal and DNA precipitation, we followed the instructions of Qiagen GenePure DNA Isolation Kit, with the addition of 2 μl glycogen (Sigma-Aldrich) to aid DNA precipitation. We then suspended the purified DNA in 80 μl of Qiagen DNA hydration solution and measured DNA concentration using a Qubit 2.0 (Invitrogen). Libraries were prepared using Kapa PCR-free kits and sequenced by the Quantitative Genomics Facility service platform at the Department of Biosystem Science and Engineering (D-BSSE, ETH) Basel, Switzerland, on an Illumina HiSeq 2500 sequencer. Two samples failed this sequencing step, leaving a total of 60 samples for subsequent analyses (G-33 and SK-1; table 1).

### Ecological Covariates and Subpopulation age

We summarized ecological covariates (i.e., catchment area, depth, distance to the sea, electrical conductivity, height above the sea, pH, plant cover, submersion time, and surface area) using a principal component analysis (PCA) in R v.4.0.3 (R Core Team 2020) (see Dubart et al. (2020) for more details). Measures of area and length were log_10_-transformed beforehand. The first two axes of the PCA explained 43.90% (PC1 25.39% and PC2 18.51%) of the variance and were ecologically meaningful. The first principal component described the impact of the sea or “marineness” (e.g., proximity to the sea, water chemistry [salinity, pH level], plant cover [fewer plants closer to the sea]), and represented a gradient from marine to terrestrial ponds. PC2 described geophysical properties independent of the sea (e.g., pond size, depth, catchment area) and represented a gradient from small to large ponds. The age of the subpopulation was assessed using biannual sampling data, with the maximum observed age being 31.5 years, as sampling started in 1982 (Ebert et al. 2013; Pajunen and Pajunen, 2003). A subpopulation was considered newly established if animals were observed after three consecutive visits without seeing animals, i.e., animals not being seen for more than a year. The chance of a subpopulation remaining undetected for three visits in a row was estimated to be below 2%, as the detection probability of *D. magna* is 0.74 in this survey (Dubart et al. 2020). The subpopulation age was log_10_(age + 1)-transformed for statistical analyses. The geographical distances between ponds were calculated using the R package *geodist* v.0.0.7 (Padgham and Sumner 2019) and log_10_-transformed for statistical analyses. Infection status with the locally common microsporidian parasite *Hamiltosporidium tvaerminnensis* (Haag et al. 2011), another ecological factor that may explain genomic diversity in the focal metapopulation (Cabalzar et al. 2019), was determined based on field records and on the presence of *H. tvaerminnensis*-specific sequencing reads in our pool-seq samples.

### Mapping Genomic Reads and Variant Calling

Raw reads were assessed for quality with FastQC v.0.11.8 (http://www.bioinformatics.babraham.ac.uk/projects/fastqc) and subsequently trimmed to remove low-quality sequence and adapter contamination using the default setting on Trimmomatic v.0.39 (Bolger et al. 2014). The second run of FastQC confirmed successful trimming. These trimmed, paired-end reads were interleaved with seqtk v.1.2 mergepe (https://github.com/lh3/seqtk). The *D. magna* XINB3 individual genome (BioProject ID: PRJNA624896; Fields et al., in prep.) was used as the reference genome when mapping interleaved reads with bwa-mem2 v.2.2.1 (Vasimuddin et al. 2019). Because this reference genome originates from a genotype collected from the same metapopulation, it is closely related to the metapopulation samples in our study. SAMtools v.1.7 (Li et al. 2009) was used to convert SAM files to BAM files, coordinate-sort individual BAM files, and remove unmapped reads. Read groups were added, and duplicates were marked for individual BAM files using the Picard Toolkit v.2.23.9 (Broad Institute 2020). The average read depth was estimated using SAMtools function depth. INDELs were realigned with GATK v.3.8 RealignerTargetCreator (McKenna et al. 2010; Van der Auwera et al. 2013), which created target intervals, and GATK IndelRealigner. Variants were called using GATK UnifiedGenotyper. These analyses were conducted at the sciCORE (http://scicore.unibas.ch/) scientific computing center at the University of Basel using a snakemake (Mölder et al. 2021) workflow. The VCF file was filtered to include high-quality (QUAL > 30, MQ > 40, QD > 2.0, FS < 60) sites with biallelic SNP variants (i.e., excluding INDEL variants) using vcffilter from the C++ library vcflib v.1.0.0_rc2 (Garrison et al. 2021) and VCFtools v.0.1.16 (Danecek et al. 2011). Depth estimates for each sample at each site were recalculated based on the allelic depths using VcfFilterJdk v.1f97a34 (Lindenbaum and Redon 2018), as GATK includes uninformative reads in the depth estimate but does not include them in the allelic depth estimates. Afterward, we masked genotypes with a depth of coverage (DP) less than ten using VCFtools and BCFtools v.1.9 (Danecek et al. 2021), and we masked genotypes with allele depth (Ad) of the minor allele equal to 1 using VcfFilterJdk. By applying this minor allele read count filter for each individual sample, we chose a filter that is DP-aware, and thus, more conservative than the more commonly used minor allele frequency (MAF) filters in pool-seq studies to avoid sequencing errors (Gautier et al. 2013).

### Sequence Variation and Population Genetic Analyses

Overall, synonymous and nonsynonymous genomic diversities were estimated as π, π_S_, and π_N_, respectively, using SNPGenie v.1.0 (Nelson et al. 2015). Because SNPGenie makes calculations per contig, we split the reference FASTA, annotation, and VCF files into individual contigs using *PopGenome* v.2.7.2 (Pfeifer et al. 2014). We converted the split annotation files to GTF format using GffRead v.0.12.1 (Pertea and Pertea 2020). We used π_S_ as a proxy for effective population size (Leroy et al. 2021) and tested for the association between π_S_ and pond volume (a rough proxy for population size) and the number of mitochondrial haplotypes (a rough proxy for the number of founders and immigrants). We estimated pond volume as a pyramid based on depth and surface area. We estimated the number of mitochondrial haplotypes by reconstructing them from the trimmed sequencing data, starting with mapping interleaved reads to the mitochondrial reference sequence (V3.1; Fields et al., in prep.) using bwa-mem2. We then used the resulting BAM files as input for *RegressHaplo* v.0.1 (Leviyang et al. 2017) in R to reconstruct haplotypes of all samples individually with default parameters. Specifically, we subset BAM files using BEDtools v.2.30.0 (Quinlan 2014) to focus on a genetic region (from position 10,800 to 12,800) without long conserved regions to avoid performance issues (Leviyang et al. 2017). For these calculations made in R, we used the package data.table 1.13.6 (Dowle and Srinivasan 2020) and log_10_-transformed estimates of π_S_, pond volume, and the number of mitochondrial haplotypes. We used the R package *poolfstat* v.2.0.0 (Gautier et al. 2022) to estimate pairwise genomic differentiation based on the Ad in the VCF file. We removed sites with missing genotypes, converted the pooldata object generated with *poolfstat* to allele frequencies, and then conducted PCA with the *pcadapt* v.4.3.3 (Privé et al. 2020) R package and t-SNE using *Rtsne* v.0.15 (Krijthe 2015) R package with 5D retained from the initial PCA step, perplexity 19, and 5,000 iterations. For generating the input for GESTE v.2 (Foll and Gaggiotti 2006), which is a Bayesian method based on the *F*-model to estimate *F*_ST_ of subpopulations and to relate *F*_ST_ to environmental factors using a generalized linear model, we used *poolfstat*'s function pooldata2genobaypass() to convert the VCF file to allele read counts. After conversion, we corrected the corresponding haploid pool sizes as described in Feder et al. (2012) using a modified version of the script baypass2bayescan.py (Stern and Lee 2020).

### Associations Between Covariates, Genomic Diversity, and Genomic Differentiation

To find associations between genomic diversity and ecological covariates (i.e., subpopulation age, PC1, PC2, mean distance to the two nearest neighbors (NN2), *H. tvaerminnensis* infection status), we performed a multiple regression analysis using a type two ANOVA from the *car* v.3.0–10 (Fox and Sanford 2019) R package. Using the common and simple measures for the isolation of NN2 led to the same result as using other measures of isolation (such as NN1 to NN7; supplementary fig. S1, Supplementary Material online). Additionally, after observing island-specific clusters in the dimensionality-reduction analysis of our genomic data, we also included the island of origin as a factor in a second model. We ran the Bayesian GESTE methodology with the same covariates and separately checked for associations between pairwise genomic differentiation and each ecological covariate (i.e., geographic distance, mean subpopulation age, mean PC1, mean PC2, and mean NN2) using distance-based Moran's eigenvector maps (dbMEM) analysis by redundancy analysis (RDA) to test for IBD, isolation-by-environment, or age-specific genomic differentiation. RDAs were performed on the overall data and separately on data from each island. Specifically, we separately transformed each explanatory variable into dbMEMs using the R package *adespatial* v.0.3–14 (Dray et al. 2021) and decomposed the response variable, pairwise *F*_ST_, into principal components using the R base *stats* function prcomp(). The RDAs were done in R using the package *vegan* v.2.5–7 (Oksanen et al. 2020), with significance assessed by 1,000 permutations. Finally, we tested for a correlation between genomic diversity and the ecological variables underlying the PCA for habitat.

### *Daphnia Sinensis* Genome Annotation

To estimate (non)synonymous divergence and the rate of (non)adaptive nonsynonymous substitutions, we downloaded the available *D. sinensis* genome (ASM1316709v1; GenBank accession: GCA_013167095.1) from NCBI and RNA-seq reads (run accessions: SRR10389290, SRR10389293, and SRR10389294) from EMBL. *Daphnia sinensis* is closely related to *D. magna* (Cornetti et al. 2019). We removed adapters from the reads using fastp v.0.20.0 (Chen et al. 2018) and checked its success with FastQC. To annotate the genome, we used MAKER2 v.2.31.10 (Holt and Yandell 2011). Specifically, we created a database from the *D. sinensis* genome and individually aligned the trimmed RNA-seq reads to it using STAR v.2.7.4a (Dobin et al. 2013). The database was used to generate reference-assisted transcriptomes with Trinity v.2.12.0 (Grabherr et al. 2011). We checked biological completeness using BUSCO v.3.0.2 (Seppey et al. 2019) and the arthropoda_odb9 gene set (creation date: February 7, 2017). Whereas we obtained target proteins by applying TransDecoder v.5.5.0 (https://github.com/TransDecoder/TransDecoder) on the transcriptomes, we used diamond v.2.0.11.149 (Buchfink et al. 2021) as well as hmmer v.3.3.2 (hmmer.org) to find matches of the ORFs to swissprot and pfam. Pyfasta v.0.5.2 (https://github.com/brentp/pyfasta/) was used to split intermediate FASTA file outputs. To obtain transcript hints, the individual transcript files were concatenated and mapped using minimap2 v.2.22-r1105 (Li 2018) before collapsing isoforms using collapse_isoforms_by_sam.py from the Cupcake tool (https://github.com/Magdoll/cDNA_Cupcake).

### Summary Statistics of the Divergence Data

We repeated the methodology for read mapping and VCF file preparation using the *D. sinensis* genome as a reference. The rate of adaptive substitution, *α*, was calculated for each sample separately based on the total counts of (non)synonymous polymorphisms, *P*_n_ and *P*_s_, and substitutions, *D*_n_ and *D*_s_, as 1—(*D*_s_*P_n_)/(*D*_n_*P_s_) (Smith and Eyre-Walker 2002) using SNPGenie. Only substitutions without a polymorphism at the same site were counted. To calculate the rate of (non)adaptive nonsynonymous substitutions, *ω_NA_* and *ω_A_*, we estimated the number of (non)synonymous substitutions per site, d_N_ and d_S_, using SNPGenie. Afterward, we calculated *ω_A_* as *α*(d_N_/d_S_) and *ω_NA_* as (1-*α*)(d_N_/d_S_). We tested whether *ω_A_* and *ω_NA_* were correlated with *N_e_* using Spearman correlations. *N_e_* was approximated with genomic diversity at synonymous sites, π_S_, using SNPGenie.

### Empirical Data of Single, Large, Stable Population

To compare our focal metapopulation with a single, larger, more stable *D. magna* population, we estimated overall genomic diversity, π, genomic diversity at (non)synonymous positions, π_N_, and π_S_, (non)synonymous divergence, d_N_ and d_S_, and the rate of (non)adaptive nonsynonymous substitutions, *ω_NA_* and *ω_A_*, for the *D. magna* population from the large Aegelsee lake near Frauenfeld, Switzerland (47°33′28.0″ N, 8°51′46.0″ E; surface area around 30,000 m^2^). This population is at least 60 years old and has an estimated minimum population size of over 10 million individuals. The Aegelsee does not entirely freeze in winter nor dry up in summer. However, fall and winter conditions result in little to no overwintering of *D. magna* (Ameline et al. 2021). In spring, *D. magna* hatch from resting eggs (Ameline et al. 2022). We collected a sample of 102 individuals in the spring of 2017, pool-sequenced, and prepared them for analysis identically to the metapopulation samples. To calculate the genomic summary statistics, we used SNPGenie with two separate reference genomes, i.e., *D. magna* and *D. sinensis*.

### Simulations

To further investigate relationships between (non)synonymous genomic diversity and effective population size in this metapopulation, we simulated different-sized populations and compared their variation in (non)synonymous genomic diversity with estimates from our collected natural subpopulations. Using a nonWright–Fisher model in SLiM v.3.6.0 (Haller and Messer 2019), we simulated a 100 kilobase pair stretch of coding DNA in panmictic populations of sizes 100, 200, 300, 400, and 500 with default recombination rate of 1 × 10^−8^ and a mutation rate of 1 × 10^−8^, which has previously been estimated from *D. magna* clones collected in or near the focal metapopulation (Ho et al. 2020). We simulated asexually reproducing individuals with sexual reproduction—only every eighth generation, as we estimate to be the case during the summer season (May to September)—at the study site. We ran the simulations until populations reached mutation-drift equilibrium, which is approximately ten times as many generations as the size of the population (Haller and Messer 2019). We made simulations with different distributions of the selection coefficient, i.e., distribution of fitness effects (DFE), for nonsynonymous mutations for each population size. The DFE was either fixed at zero or drawn from a gamma distribution (mean = −0.03 and shape = 0.2 or mean = −0.05 and shape = 0.5). However, we fixed the selection coefficient of synonymous mutations at zero for all runs. We conducted 1,000 replicate simulations for each setting. To be able to compare the results of our simulations with more conventional sexual systems, we repeated the same simulations but with solely sexually reproducing individuals.

To compare the observed (non)synonymous substitution rates in the metapopulation to in silico data, we performed a second set of simulations with substitution tracking enabled. We increased the number of generations per simulation to 100,000 and the simulated sequence length to one Mbp to get a significant number of polymorphisms that would reach fixation. Moreover, 1.5% of the nonsynonymous mutations were beneficial (*s* = 0.0001), as reported for *Drosophila* (Huber et al. 2017), making the calculation of *ω_A_* and *ω_NA_* more meaningful.

## Results

### Sequencing and Population Structure

**Fig. 2.**
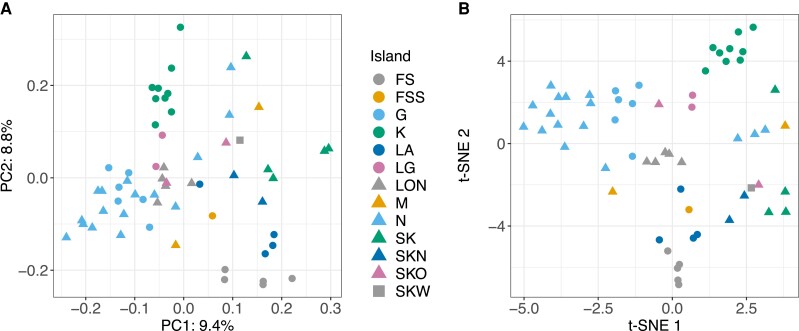
Dimensionality-reduction using PCA and t-SNE. PCA (*A*) and t-SNE (*B*) reveal spatial population structure based on whole-genome allele frequency data. Samples from the same island usually form clusters. Colored symbols represent the island of origin (see legend). Percentages in (*A*) give the amount of variance explained by PC1 and PC2.

A total of 60 *D. magna* subpopulations distributed throughout almost the entire survey area of the metapopulation were successfully sequenced (fig. 1 and table 1). Between 72% and 99% of our pool-seq reads were mapped to the *D. magna* reference genome (supplementary table S1, Supplementary Material online). Samples with a low mapping percentage were infected with a locally common microsporidian parasite (*Hamiltosporidium tvaerminnensis*; supplementary table S1, Supplementary Material online) that can dominate the sequencing reads when whole genomes of host and parasites are cosequenced (Angst et al. 2022). With only a few exceptions, the average read coverage for most *D. magna* samples was beyond 20× (supplementary table S1, Supplementary Material online). After variant filtration, we were left with 1,540,716 SNPs to analyze. A PCA of this SNP data indicated an overall structure in the data (fig. 2*A*). As expected, most samples originating from the same island clustered together. The PCs reflected geographic distribution to some degree, with longitude being positively correlated with PC1 (Spearman's *r*(58) = 0.71, *P* < 0.001) and latitude negatively correlated with PC3 (Spearman's *r*(58) = −0.50, *P* < 0.001). The clustering by island became more defined when samples were pinpointed on a 2D map based on the first five PCs using t-distributed stochastic neighbor embedding (t-SNE; fig. 2*B*). Interestingly, in some cases, samples from some islands fell into multiple subclusters (LA, M, and N), reflecting distinct regions on the islands (supplementary fig. S2, Supplementary Material online). These subclusters may represent different colonization histories for these regions. Furthermore, samples from island SK and its close neighboring islands, SKN, SKO, and SKW, formed one cluster, so did samples from three geographically close islands FS, FSS, and LA (fig. 2*B*).

### (Non)Adaptive Genomic Divergence

**Fig. 3.**
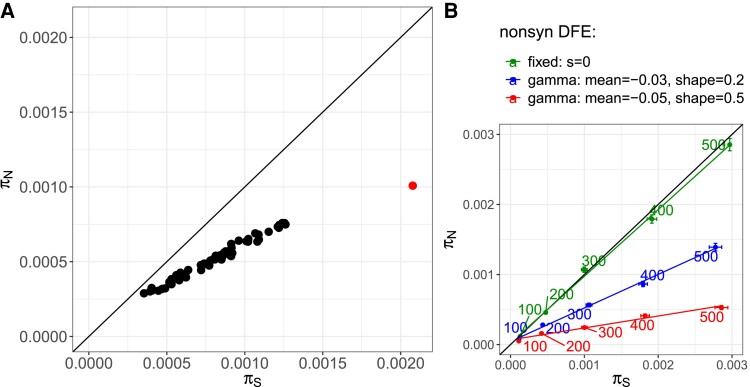
Association between nonsynonymous, π_N_, and synonymous, π_S_, genomic diversity in populations of different sizes. (*A*) π_N_ plotted against π_S_, with each dot representing one (sub)population. The slope of the data cloud is smaller than one (0.52). Thus, more diverse subpopulations have relatively fewer nonsynonymous polymorphisms, suggesting that purifying selection is stronger in these subpopulations. The single dot to the right is the estimate for the single, large, and stable population from Switzerland. (*B*) Simulated (non)synonymous genomic diversity using SLiM (Haller and Messer 2019). The colored numbers show the simulated population sizes. π_S_ correlates well with *N_e_*. Colors indicate the nonsynonymous distribution of fitness effects (DFE) following different gamma distributions or fixed to zero (total absence of selection; *s* = 0). The DFE following a gamma distribution with a mean of −0.05 and a shape of 0.5 is the scenario with the strongest selection. Error bars indicate the standard error around the mean of 1,000 runs. Colored lines are fits for the different nonsynonymous DFEs using linear regressions. The diagonal line indicates the one-to-one ratio in both plots.

**Fig. 4.**
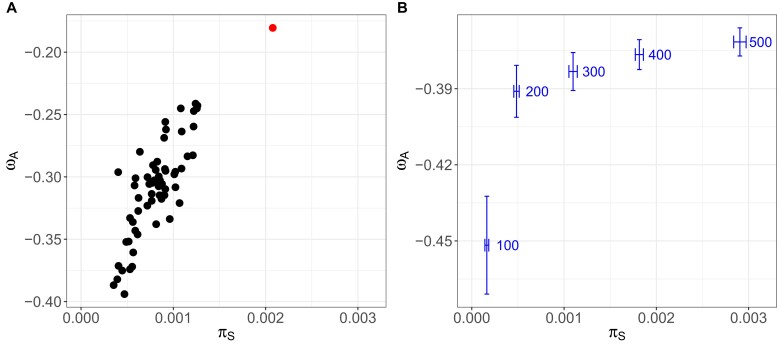
Association between the proportion of adaptive nonsynonymous substitutions, *ω_A_*, and synonymous genomic diversity, π_S_, in differently sized populations. (*A*) Observed *ω_A_* for each subpopulation; *ω_A_* is positively associated with π_S_, which is a useful approximation for *N_e_*. The single dot at the top right shows the estimate for the single, large, and stable population from Switzerland. (*B*) Shows the positive association of *ω_A_* with π_S_ and the simulated population size. Horizontal and vertical error bars indicate the standard error around the mean of 1,000 runs (simulations were performed with SLiM (Haller and Messer 2019)).

**Fig. 5.**
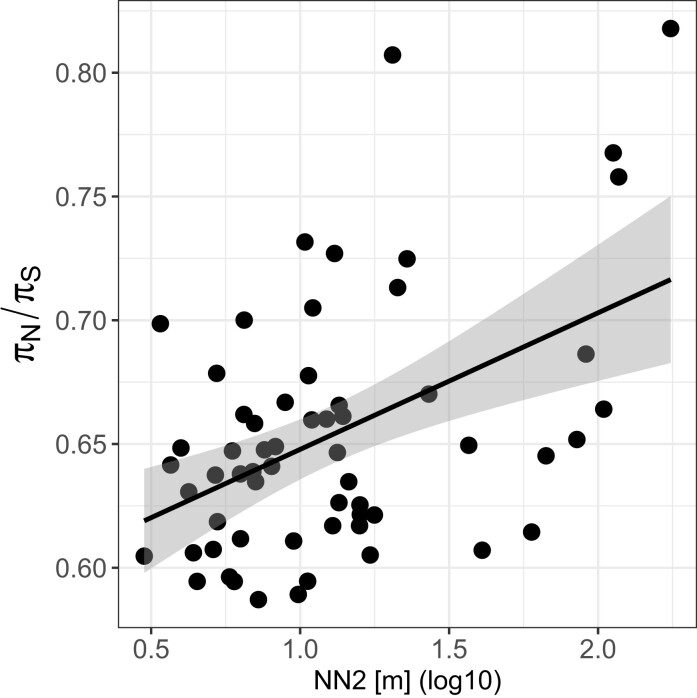
π_N_/π_S_ depends on the degree of spatial isolation of a subpopulation. The number of nonsynonymous polymorphisms relative to synonymous polymorphisms correlates positively with the isolation measure, NN2 [m]. A positive correlation was found using a Spearman correlation test (*R* = 0.36, *P* = 0.005). Each dot represents one subpopulation. The regression line is from a linear model with the 95% confidence interval depicted as shading around the line to visualize the positive correlation.

Synonymous genomic diversity, π_S_, can be used to approximate the theoretical quantity *N_e_*, acting as a stand-in to predict how populations will behave evolutionarily. This is because π_S_ is determined by mutation rate and *N_e_*. Since the mutation rate is similar across subpopulations, the observed differences in their π_S_ are largely due to their variation in *N_e_*. The smaller the *N_e_*, the stronger the genetic drift, and thus, the faster the loss of mutations (Wright 1931). We tested this approximation by correlating π_S_ with pond volume (assuming larger ponds have larger populations and are more likely to receive immigrants) and with the number of mitochondrial haplotypes (assuming the number of haplotypes is representative of the number of colonists and immigrants). While both variables showed a positive association, as expected, it was not a strong correlation: the correlation of π_S_ with pond volume was Pearson's *r*(58) = 0.25, *P* = 0.05, whereas the correlation of π_S_ with mitochondrial haplotypes was *F*(1,58) = 4.502, *P* = 0.038, and *R*^2^ = 0.07. The weak correlations are not entirely unexpected because the degree to which *N_e_* correlates with population census size, *N*, is highly variable and depends on a range of ecological and evolutionary details in an individual biological system (Waples 2022). For example, in this metapopulation, large subpopulations can be founded by one or several individuals that have undergone clonal expansion. Observed nonsynonymous and synonymous genomic diversity, π_N_ and π_S_, were strongly positively correlated, but with a slope of less than one (fig. 3*A*, supplementary fig. S3, Supplementary Material online). More diverse subpopulations showed larger departures from a one-to-one ratio, meaning that π_N_ decreased relative to π_S_ as the diversity of subpopulations increased (fig. 3*A*, supplementary fig. S3, Supplementary Material online). We found the same relationship between nonsynonymous and synonymous diversity when we drew nonsynonymous mutations from a distribution of negative selection coefficients to simulate partially asexual (fig 3*B*) and sexual (supplementary fig. S4, Supplementary Material online) populations of different sizes. When the selection coefficient of all mutations is zero, the slope is one (fig. 3*B*). Therefore, observed and simulated results coincided with expectations from population genetic theory, that purifying selection is more efficient in removing deleterious nonsynonymous polymorphisms from populations with higher synonymous diversity (populations with higher *N_e_*) (Charlesworth 2009). Furthermore, the ratio of nonsynonymous to synonymous genomic diversity in the subpopulations, π_N_/π_S_, was positively correlated with the isolation measure (mean distance to the two closest neighboring subpopulations, or NN2) (Spearman's *r*(58) = 0.36, *P* = 0.005; fig. 5), suggesting that purifying selection is less efficient in more isolated (= less diverse) subpopulations. Positive selection, measured as the rate of adaptive nonsynonymous substitutions, *ω_A_*, was higher in populations with higher synonymous diversity, a pattern also seen in the data from the metapopulation estimated with the outgroup *D. sinensis* (fig. 4*A*, supplementary results, Supplementary material online) and in the simulation results (fig. 4*B*). This supports that higher synonymous diversity is an approximation for higher effective population size allowing for more efficient selection. Because natural selection and genetic drift are nonindependent, positive selection may be more efficient, as it is less affected by genetic drift. (The rate of nonadaptive nonsynonymous substitutions, *ω_NA_*, is an inversion of *ω_A_* and was lower in larger populations [supplementary figs. S5 and S6, Supplementary Material online]).

### Genomic Diversity in Relation to Ecology

**Fig. 6.**
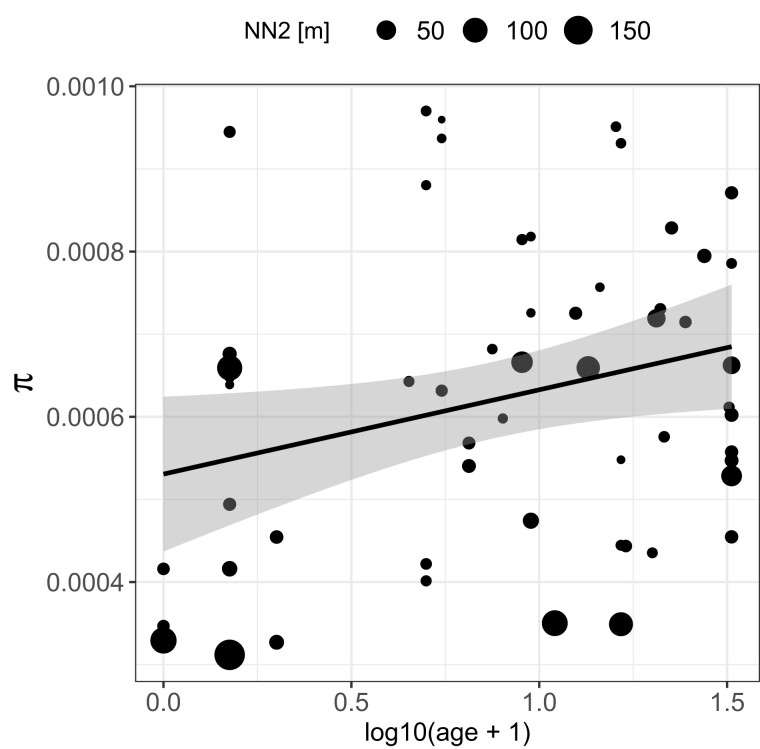
Genomic diversity, π, can be statistically explained by subpopulation age (x-axis) and isolation level (NN2, larger size of symbol for more isolated populations) (compare table 2). Genomic diversity within subpopulations is positively correlated with subpopulation age (positive slope of regression line) and negatively correlated with the isolation measure, NN2 [m] (symbol sizes become smaller towards the top of the graph). Population age is log_10_(age + 1)-transformed. Each dot represents a subpopulation. The regression line is based on a linear model (π ∼ log_10_(age + 1)) with the confidence interval depicted as shading around the line.

Genomic diversity, π, was estimated separately for each *D. magna* subpopulation. It ranged by nearly an order of magnitude from 3 × 10^−4^ to 1 × 10^−3^ with a mean of 6 × 10^−4^. We tested whether genomic diversity was associated with subpopulation age since colonization, NN2, ecological variables, and infection status. Using multiple regression analysis, we found that young, isolated subpopulations were less diverse than older, less isolated subpopulations (table 2 and fig. 6, supplementary fig. S7, Supplementary Material online). These findings held true after correcting for the island of origin (supplementary table S2, Supplementary Material online). We found no significant association between π and either PC1 (marineness), PC2 (pond geometry), or *H. tvaerminnensis* infection status in the overall model (table 2). However, we found a slight correlation between genomic diversity and mean pH (Pearson's *r*(58) = 0.30, *P* = 0.02), when testing the ecological variables underlying the habitat PCA separately. This became nonsignificant when correcting for multiple testing, which was necessary because several ecological variables underlying the habitat PCA were tested.

**Table 2.** Type two Analysis of Variance Between Genomic Diversity, π, and Explanatory Variables.

Explanatory Variable	df	Statistics	*P*	*R* ^2^
Population age	54	*t* = 2.02	**0**.**048**	0.308
PC1 (marineness)	54	*t* = 1.13	0.265	
PC2 (pond geometry)	54	*t* = −0.52	0.605	
NN2 (isolation)	54	*t* = −3.152	**0**.**003**	
Infection status	1/54	*F* = 0.5202	0.474	

This type of analysis of variance follows the principle of marginality, testing each term after all others (partial sum of squares model). One *R*^2^ value is given for the complete model. Significant associations are in bold.

### Genomic Differentiation in Relation to Ecology

**Fig. 7.**
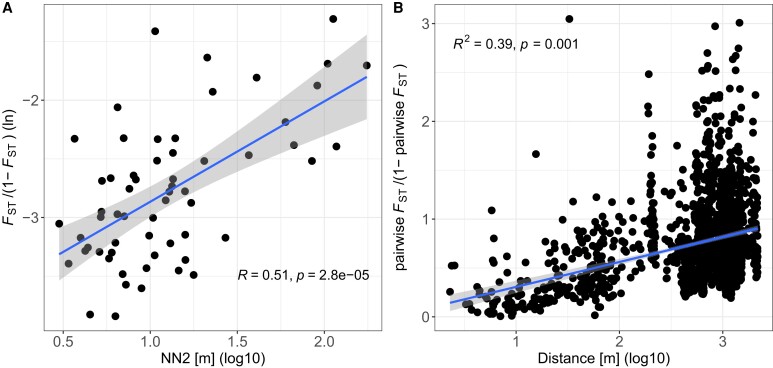
Isolation by distance (IBD). (*A*) *F*-model-based *F*_ST_ positively correlates with the isolation measure NN2 [m]. Each point represents one subpopulation. A positive correlation was found using a Spearman correlation test (see statistics within the graph). (*B*) Pairwise population differentiation correlates positively with geographic distance. Each point represents the comparison between two subpopulations. A positive correlation was found using dbMEM analysis by RDA (see statistics within the graph). In both plots, the blue line is a regression line based on a linear model with the confidence interval depicted as shading around the line to visualize the positive correlation.

Genomic differentiation between subpopulations, as estimated with *F*-model-based *F*_ST_ using GESTE, had a mean of 0.06 and a range from 0.02 to 0.21. GESTE's test for association with subpopulation age, environment, and isolation suggested that genomic differentiation was best explained by a model with only the measure of isolation, NN2, i.e., the model with the highest posterior probability (postProb = 0.48; table 3 and fig. 7*A*, supplementary fig. S8, Supplementary Material online). Models without this isolation measure had a posterior probability of essentially zero, rendering them unlikely. The relationship between *F*_ST_ and NN2 remained significant when we corrected for the island of origin and infection status (*F*(1,45) = 67.961, *P* < 0.001, and *R*^2^ = 0.75). Furthermore, as estimated using *poolfstat*, pairwise genomic differentiation correlated with mean NN2 (dbMEM analysis by RDA: *R*^2^ = 0.05, *P* = 0.013; supplementary fig. S9, Supplementary Material online) but the explained variation is low. Therefore, both the *F*-model and the pairwise approach for estimating genomic differentiation found that geographically isolated subpopulations showed greater differentiation than less isolated subpopulations, and that the geographical isolation of subpopulations was the primary driver among the factors tested for overall patterns of genomic differentiation in the focal metapopulation. Additionally, pairwise genomic differentiation and geographic distance were positively correlated (*R*^2^ = 0.39, *P* = 0.001; fig. 7*B*), meaning that subpopulations separated by greater geographical distance were more differentiated than geographically closer subpopulations, i.e., IBD. We also found IBD when looking specifically at combinations of subpopulations within islands (mean *R*^2^ = 0.38). Correlations between pairwise genomic differentiation and the remaining candidate variables (mean of each PC1 (marineness), PC2 (pond geometry), and subpopulation age) were nonsignificant. This pattern corresponds with results from the GESTE, pointing to IBD as the dominant pattern. On the other hand, the immediate effect of genetic bottlenecks during population founding on higher population differentiation among young subpopulations is not as strong in the overall analysis. However, the finding by Haag et al. (2005) that newly founded subpopulations are more differentiated from each other than older subpopulations was confirmed when looking specifically at comparisons between the age class of newly founded subpopulations and older age classes (≤2 years old: mean *F*_ST_ = 0.49, CI95 = 0.47–0.51; >2 and ≤15 years old: mean *F*_ST_ = 0.35, CI95 = 0.33–0.37; >15 years old: mean *F*_ST_ = 0.36, CI95 = 0.35–0.38; Wilcoxon's *W* = 21,774, *P* < 0.001 and *W* = 22,103, *P* < 0.001 for old and intermediate age classes vs. young age class, respectively). The finding that intermediate and old age classes did not differ in mean pairwise *F*_ST_ (Wilcoxon's *W* = 28,697, *P* = 0.16) reflects the nonsignificant correlation between pairwise population differentiation and mean age and agrees with Haag et al. (2005).

**Table 3.** Associations Between Differentiation in Subpopulations, *F*_ST_, and Explanatory Variables Calculated Using GESTE.

Explanatory Variable(s)	postProb(model)
Constant	0.000
Constant, age	0.000
Constant, PC1	0.000
Constant, PC1, age	0.000
Constant, PC2	0.000
Constant, PC2, age	0.000
Constant, PC2, PC1	0.000
Constant, PC2, PC1, age	0.000
Constant, NN2	0.478
Constant, NN2, age	0.012
Constant, NN2, PC1	0.027
Constant, NN2, PC1, age	0.002
Constant, NN2, PC2	0.374
Constant, NN2, PC2, age	0.001
Constant, NN2, PC2, PC1	0.094
Constant, NN2, PC2, PC1, age	0.003

GESTE is a Bayesian method based on the *F*-model for estimating the *F*_ST_ of subpopulations and for testing associations between *F*_ST_ and explanatory variables. Here, age, PC1, PC2, and NN2 are explanatory variables. Each combination of explanatory variables in the first column is accompanied by a posterior probability in the second column. Unrounded posterior probabilities sum to one. Models without NN2 result in a posterior probability of 0.

### Single, Large, Stable Population

To compare our focal metapopulation's genetic summary statistics with a single large population of the same species, we used pool-seq data from the relatively large Aegelsee *D. magna* population in Switzerland. Based on the analysis of 1,056,626 SNPs, we estimated a π value of 1.5 × 10^−3^, about 2.5 times higher than that estimated for the entire metapopulation. The estimates of π_N_ (1.0 × 10^−3^) and π_S_ (2.0 × 10^−3^) corresponded with our expectations for a larger population, as they showed higher π_S_ and more deviation from the one-to-one line than the metapopulation samples (fig. 3*A*). Also, *ω_A_* (−0.18) and *ω_NA_* (0.32) were higher and lower than in the metapopulation samples, respectively, as expected for a larger population (fig. 4, supplementary fig. S5, Supplementary Material online).

## Discussion

Extinction–(re)colonization dynamics are a key aspect in distinguishing dynamic metapopulations from larger, more stable populations with gene flow (Hanski 1999; Wang and Altermatt 2019). Understanding how these metapopulation dynamics influence molecular evolution is a step toward understanding how metapopulations differ from the more extensively studied Wright–Fisher populations. This study presents evidence that nondeterministic processes play a large role in a highly dynamic, natural metapopulation, and that subpopulation founding with initial genetic bottlenecks leads to low genomic diversity as assumed under the propagule model. This model predicts strong genetic drift, a weakened efficacy of selection, and the accumulation of deleterious mutations, all of which we observed here, in the studied metapopulation. Contrasting this metapopulation with a stable, large population of the same species shows that genomic diversity is considerably lower and genetic drift is much stronger in the metapopulation.

### Evolutionary Model

By identifying the genomic variation, age, ecology, and geography of individual subpopulations, we investigated the evolution of interconnected subpopulations of a metapopulation. Previous research in our focal *D. magna* metapopulation has found high turnover dynamics, small numbers of subpopulation founders, and the accumulation of deleterious mutations in the mitochondrial genome, all consistent with the propagule model (Altermatt and Ebert 2010; Dubart et al. 2020; Ebert et al. 2013; Fields et al. 2018; Zumbrunn 2011). Subpopulations tend to be short-lived, undergoing frequent and strong genetic bottlenecks during the colonization of empty habitat patches and subsequently suffering from a genetic load. The effective population size of subpopulations is correspondingly low (Walser and Haag 2012), and genetic drift is a strong evolutionary force that can reduce natural selection efficacy. Our study corroborated these features, demonstrating low genomic diversity and high differentiation among subpopulations, particularly young ones. For most genomic summary statistics, isolation from neighboring subpopulations was the most important factor driving variation, suggesting that, especially in remote parts of the metapopulation, there is moderate to low gene flow among subpopulations. In more isolated subpopulations, we found lower synonymous genomic diversity, π_S_, —a proxy for *N_e_*—(fig. 5), and in subpopulations with lower π_S_, we found higher rates of nonadaptive nonsynonymous substitutions, i.e., deleterious substitutions (supplementary fig. S5, Supplementary Material online). Our evolutionary model of the *D. magna* metapopulation contrasts markedly with a much larger and older population of the same species, which was considerably more diverse and showed more efficient purifying selection, likely due to a weaker genetic drift. These results confirm the theory of lower adaptive evolution in metapopulations (Whitlock 2004).

### Accumulation of Deleterious Mutations

It has been shown that *N_e_* explains cross-species variation in the rate of molecular evolution (Eyre-Walker 2006; Eyre-Walker and Keightley 2009; Gossmann et al. 2012). The within-species analog of this prediction is that in populations of different sizes, as they are commonly found in metapopulations, differences in genomic diversity are expected to explain variation in the rate of molecular evolution among (sub)populations. Populations with lower *N_e_* are expected to accumulate deleterious mutations faster, thus displaying higher *ω_NA_*. Consequently, their rate of adaptive substitution, *α*, would be lower, as shown across species by Galtier (2016). In our focal metapopulation, we found higher *ω_NA_* in subpopulations with smaller π_S_ (a proxy for lower *N_e_*; supplementary fig. S5, Supplementary Material online). Consequently, *ω_A_* in subpopulations with smaller π_S_ was decreased, indicating that selection is less efficient in these subpopulations and that genetic drift may reduce the efficiency of selection. Plotting π_N_ against π_S_ shows a strong correlation, but with a slope clearly smaller than one (fig. 3). Thus, in subpopulations with lower π_S_, nonsynonymous diversity is higher relative to synonymous diversity than in subpopulations with higher π_S_ (fig. 3). This relationship might be because (1) purifying selection removes nonsynonymous deleterious mutations more efficiently in larger subpopulations, and (2) gene flow masks genetic load so that subpopulations with a lower π_N_/π_S_ ratio are less isolated (fig. 5). In the second scenario, recessive deleterious mutations could accumulate after a colonization bottleneck. Gene flow increases heterozygosity by introducing variation from less-related individuals, which would result in hybrid offspring with increased fitness, i.e., hybrid vigor (Ebert et al. 2002; Lohr and Haag 2015).

**Fig. 8.**
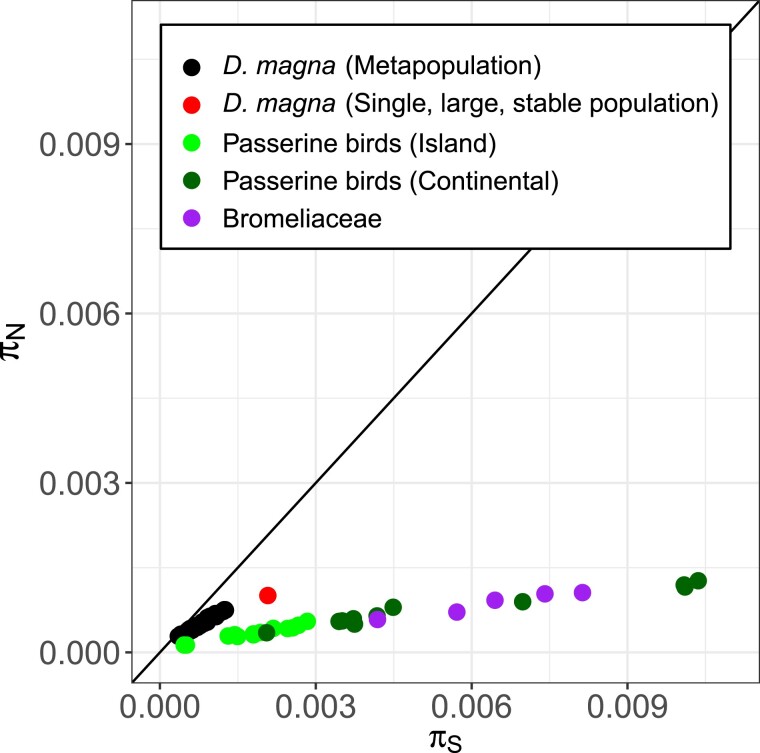
Association between nonsynonymous and synonymous genomic diversity in different systems and populations of different sizes. Our data presented in fig. 3*A* are compared with other published whole-genomic datasets: Passerine birds originating from island and mainland populations (Leroy et al. 2021) and different Bromeliaceae species (Yardeni et al. 2022). The relationship between π_N_ and π_S_ is the same in all systems. In passerine birds, small populations (on islands) are less diverse than large populations (on mainland) which we also observed in the *D*. *magna* system.

We contrast our findings with previous studies using whole-genome data to explore the relationship between π_N_ and π_S_, mainly between species. Leroy et al. (2021) showed the effect of *N_e_* on (non)synonymous diversity and (non)adaptive evolution in insular versus continentally distributed populations of different bird species (fig. 8). Their comparison also included an insular and a continental sample from the same bird species (*Fringilla coelebs*). *N_e_* was positively associated with *ω_A_*. We undertook a similar approach within a metapopulation of a single species with subpopulations of different ages and isolation levels and found the same relationship between π_N_ and π_S_ as well as π_S_ and *ω_A_*. This suggests that π_S_ explains variation in evolution within a species just as it does across species, as previous studies have noted (Eyre-Walker 2006; Eyre-Walker and Keightley 2009; Gossmann et al. 2012). By simulating different-sized populations in silico to isolate the effects of *N_e_* on *ω_A_*, we further supported this finding, thereby obtaining the same associations between π_S_, π_N_/π_S_, and *ω* as we showed in the natural populations. Our results are also consistent with results by Fields et al. (2018), who showed that in genotypes collected from different *D. magna* metapopulations, protein-coding genes in the mitochondrial genome show enrichment in deleterious mutations compared with genotypes collected from larger and more stable populations in other parts of the species’ range, and with results by Lohr and Haag (2015), who showed that genetic load is higher in *D. magna* metapopulations than in larger and more stable populations. We observed negative *ω_A_*, the cause for which, generally, is the presence of (weakly)deleterious nonsynonymous variants, a pattern we describe in the present work. This same pattern has been described for *D. magna* in Fields et al. (2022).

A striking finding of the comparison of our results with previously published estimates for the relationship between π_N_ and π_S_, is that in all studies the datapoints fall very close to a nearly straight line. Testing this relationship for just one contig of the genome revealed a large amount of variation around this line (supplementary fig. S9, Supplementary Material online), suggesting that the large number of polymorphisms available in whole-genome studies is responsible for the good fit of the data to a line. The reason for the elevated values of nonsynonymous diversity in *D. magna* (sub)populations relative to the other species is unclear. It might be due to the partial asexual reproduction, although we did not observe large differences in our simulations (see fig. 3*B*, supplementary fig. S4, Supplementary Material online), or other specifics of their demography.

### Age and Isolation as Predictors for Genomic Diversity

The founding of subpopulations by one or several individuals and the following rapid subpopulation expansion (clonal expansion in the case of cyclic parthenogens like *Daphnia*) leads to an elevation in genetic load (Haag et al. 2005) and low genomic diversity. Over time, immigration into subpopulations will increase genomic diversity. If subpopulations have a high genetic load, the rate of effective gene flow may be elevated by hybrid vigor, as has been shown experimentally for the *D. magna* metapopulation (Ebert et al. 2002). In our focal metapopulation, we confirmed that genomic diversity increases with subpopulation age. We tested if this gain in genomic diversity is faster with a higher immigration rate, by quantifying the correlation between the mean distance to the two closest neighboring subpopulations (NN2). As expected, isolated subpopulations have indeed lower genomic diversity than less isolated ones. Without further immigration, genomic diversity may even decrease rather than increase, as diversity may be lost by drift. There were, however, not sufficient isolated subpopulations that were old enough to test this assumption.

Earlier studies speculated that larger, more stable ponds with older subpopulations could be a reservoir for genomic diversity (Haag et al. 2005; Pajunen 1986; Vanoverbeke et al. 2007); however, our analysis of the relationship between genomic diversity and the ecological characteristics of ponds does not support this prediction. Larger ponds do not differ from other ponds in their genomic diversity. We also tested a finding from previous research in this metapopulation that infection with a virulent microsporidian parasite, which occurs in more than half of all subpopulations, is associated with genomic diversity (Cabalzar et al. 2019). In our study, we do not observe this correlation. However, this earlier study focused exclusively on old subpopulations and aimed to understand the evolutionary differentiation of old subpopulations evolving with and without the parasite. Here, the inclusion of younger subpopulations may have prevented us from observing this correlation.

### Genomic Differentiation is Driven by Geographical Isolation

Genomic differentiation between subpopulations is generally high, mainly because of the high turnover dynamics in this metapopulation. These dynamics lead to frequent (re)colonization of vacant habitat patches, whereby the expectation of *F*_ST_ between newly founded populations is 0.5 (Wade and McCauley 1988). We estimated population differentiation between recently founded subpopulations at ∼0.49, close to the theoretical expectation. However, as populations get older and receive immigrants, pairwise *F*_ST_ values were expected to decrease, which is what we observed. For passively dispersed aquatic invertebrates, founder effects have been suggested as a main driver of differentiation; these include a combination of a few population founders, high population growth rates, and large population census sizes (Montero-Pau et al. 2018). Founder effects occur because many aquatic invertebrates are cyclic parthenogens, so a single individual can found and, after clonal expansion, populate the entire habitat patch. Montero-Pau et al. (2018) have shown that, in passively dispersed aquatic invertebrates, founder effects outweigh selective processes and migration. These factors were all considered equally important in the so-called monopolization hypothesis for explaining the genetic structure of aquatic invertebrates (De Meester et al. 2002). Under the monopolization hypothesis, selective processes (e.g., adaptation) might be more efficient in populations with large *N_e_* that exhibit weaker genetic drift, so that selection can hinder the immigration of deleterious alleles and residential allele frequencies are favored (Lohr and Haag 2015). In populations with large *N_e_*, local adaptation, which is often observed in strongly structured aquatic populations (Decaestecker et al. 2007; Franch-Gras et al. 2017) may, therefore, reduce the effective immigration rate of nonadapted genotypes. In the small subpopulations of our metapopulation, local adaptation has not been observed so far (Cabalzar et al. 2019; Roulin et al. 2015).

Our analysis revealed that subpopulations separated by greater distance are more dissimilar than geographically closer ones, i.e., a pattern of IBD, suggesting that gene flow is more likely between geographically closer subpopulations from the same and different islands. Data consistent with this have been reported before for the same metapopulation (Haag et al. 2006; Roulin et al. 2016). It is also consistent with the finding that dispersal distance exponentially decays, in which case, long distance colonization events are rare (Dubart et al. 2020; Pajunen 1986; Pajunen and Pajunen 2003). Alternative to this gene-flow hypothesis to explain IBD, it has been suggested for several aquatic organisms that sequential colonization can shape IBD (Montero-Pau et al. 2018). However, our long-term data on local extinction and recolonization in this metapopulation suggest that the gene flow hypothesis might explain more of the observed pattern of IBD (Dubart et al. 2020; Pajunen 1986; Pajunen and Pajunen 2003). Furthermore, the isolation measure, NN2, was the variable that best explained a subpopulation's *F*_ST_ estimated with the *F*-model approach and could be shown to correlate with pairwise *F*_ST_ (fig. 7). Finding an association between NN2 and *F*_ST_ in these two complementary approaches underlines the importance of gene flow in predicting genomic differentiation in this metapopulation (Gaggiotti and Foll 2010). In contrast to our study system, previous metapopulation studies of freshwater zooplankton presented evidence against IBD on a local scale (Martin et al. 2021; Montero-Pau et al. 2017), but system-specific differences may be important. Our study species, *D. magna*, is mainly a pond-dwelling species. Other zooplankton species may inhabit different waterbodies, such as large lakes, and different ecological factors may drive their evolution. Population genomic studies in other species are needed for proper comparisons.

## Conclusion

We studied the population genomics of a well-documented, highly dynamic metapopulation to understand if metapopulations evolve differently from a large and stable population. The obvious differences between these types of populations are that metapopulations feature extinction–(re)colonization dynamics. The genomic consequences of metapopulation ecology include recurrent bottlenecks during population founding, which can lead to high genomic differentiation between subpopulations and low genomic diversity within subpopulations. Even though *D. magna* census population sizes may be large, bottlenecks cause low genomic diversity and strong genetic drift, which in turn, reduces the efficacy of selection. This is seen as lowered rates of adaptive substitutions and elevated numbers of nonsynonymous relative to synonymous polymorphisms in the metapopulation compared with the single, large, and stable population. Our results support the expected differences between these population types and suggest that nondeterministic forces dominate the evolutionary process in dynamic metapopulations. Without the prior knowledge from the long-term survey of this metapopulation (Dubart et al. 2020; Ebert et al. 2013; Pajunen and Pajunen 2003; Walser and Haag 2012), it would be more difficult to attribute the increased genetic drift to the observed metapopulation dynamics, because other processes, e.g., fluctuations in subpopulation size could also lead to a high genetic drift (Charlesworth et al. 2003). Our study does not only provide genomic insights into a well-documented metapopulation, but we also link genomics to subpopulation ecology, and thus, unravel the evolutionary mechanisms of a metapopulation in a fragmented habitat. This provides valuable insights for conservation biology and helps to understand how metapopulations evolve differently from Wright–Fisher populations. Our findings are largely consistent with the propagule model of metapopulation evolution (Slatkin 1977) and provide a striking empirical example of such a metapopulation.

## Supplementary Material

msac264_Supplementary_Data

## Data Accessibility

Analysis scripts are available at https://github.com/pascalangst/Angst_etal_2022_MBE, allele frequencies of individual subpopulations are deposited at https://doi.org/10.6084/m9.figshare.21618303, and raw data are deposited at the NCBI SRA database (BioProject IDs PRJNA862292, PRJNA883036).
