# Correction to: Genetic Drift Shapes the Evolution of a Highly Dynamic Metapopulation

**DOI:** 10.1093/molbev/msad004

**Published:** 2023-01-19

This is a correction to: Pascal Angst, Camille Ameline, Christoph R Haag, Frida Ben-Ami, Dieter Ebert, Peter D Fields, Genetic Drift Shapes the Evolution of a Highly Dynamic Metapopulation, *Molecular Biology and Evolution*, Volume 39, Issue 12, December 2022, msac264, https://doi.org/10.1093/molbev/msac264

In the originally published PDF version of this manuscript, the axis labels of figures 4, 5, and 6 were partially incorrect.

These errors have now been corrected.

