# Supplementary material for: Genetic Drift Shapes the Evolution of a Highly Dynamic Metapopulation
Source: Mol Biol Evol. 2022 Dec 6;39(12):msac264. doi: 10.1093/molbev/msac264 (PMC9778854; doi:10.1093/molbev/msac264)
Supplement: msac264_Supplementary_Data [file msac264_supplementary_data.pdf]

Supplemental Information for:

Genetic drift shapes the evolution of a highly dynamic metapopulation

Pascal Angst[1,*], Camille Ameline[1,2], Christoph R. Haag[3], Frida Ben-Ami[4], Dieter Ebert[1], and Peter D. Fields[1]

[1] Department of Environmental Sciences, Zoology, University of Basel, Basel, Switzerland

[2] Instituto Gulbenkian de Ciência, Oeiras, Portugal

[3] CEFE, Université de Montpellier, CNRS, EPHE, IRD, Montpellier, France

[4] George S. Wise Faculty of Life Sciences, School of Zoology, Tel Aviv University, Tel Aviv, Israel

[*] Corresponding author: pascal.angst@unibas.ch

Supplemental Results

D. sinensis genome annotation

Annotation of the *D. sinensis* genome resulted in nearly 21,000 gene models. The observed number of gene models in *D. sinensis* is lower than the number of gene models in the focal species, *D. magna*, though the number is not so different from the number of gene models found in other *Daphnia* species such as *D. pulex* (around 19 thousand; Ye et al., 2017). A possible explanation for the lower number of annotated genes is the more limited RNAseq resources presently available for *D. sinensis* as compared to *D. magna*. However, the number of genes present in this *de novo* annotation, and in particular the number of identifiable orthologs between the species, is more than sufficient for the target analysis of a genome-wide summary of selection on protein-coding regions.

Figure S1: Correlation analysis between different isolation estimates (NN1 to NN7). The different estimates of isolation involving only the closest or up to seven nearest neighbours are all positively correlated with each other, indicated by the (dark)blue color.

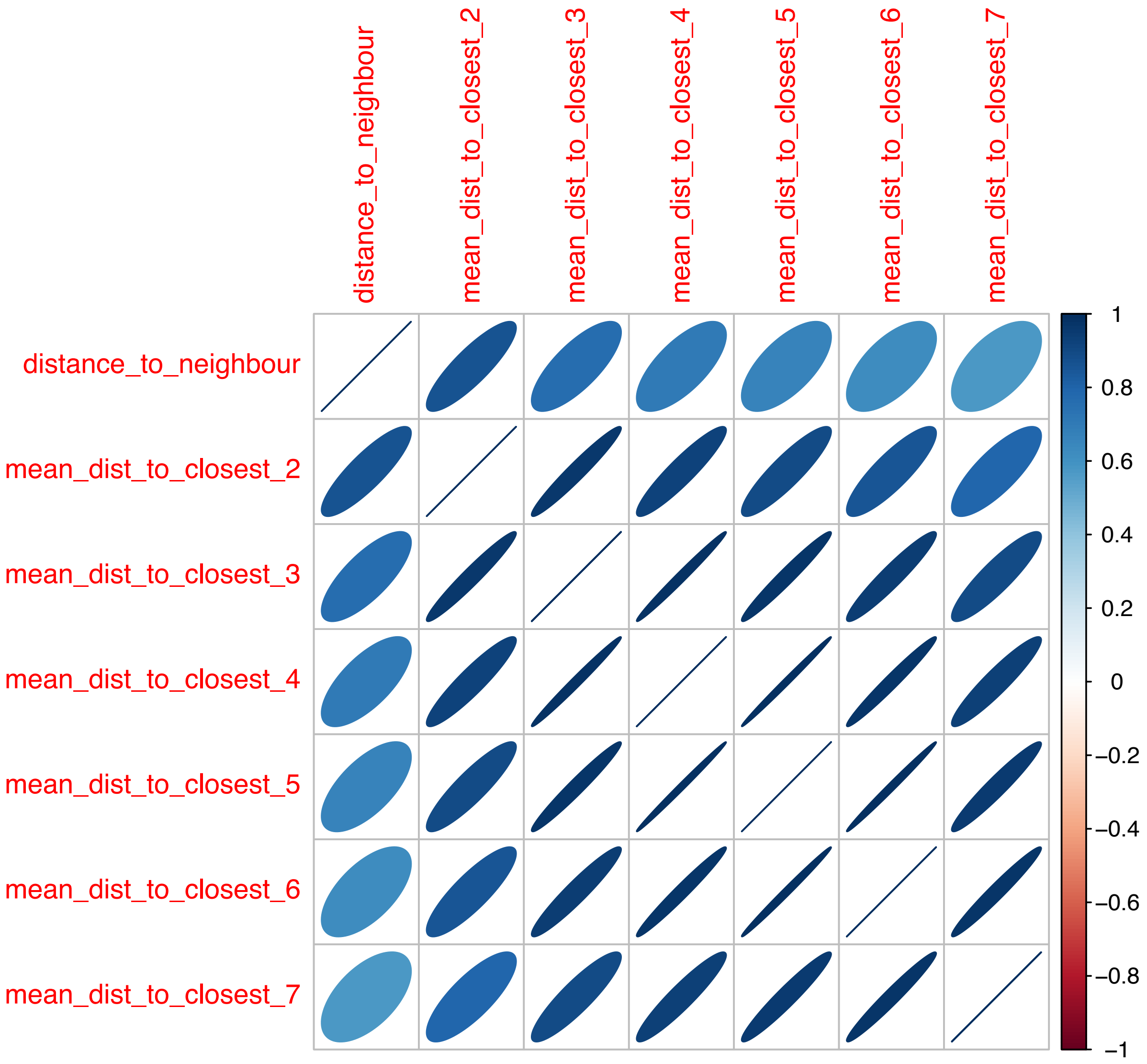

Figure S2: Dimensionality-reduction using PCA and t-SNE. This is the same figure as Figure 2 but with numbers added to ponds. PCA (A) and t-SNE (B) based on whole-genome allele frequency data reveal spatial population structure. Samples from the same island form clusters in most cases (see Figure 1). A combination of colors and symbols indicates each island of origin. The ponds on the same island are numbered consecutively.

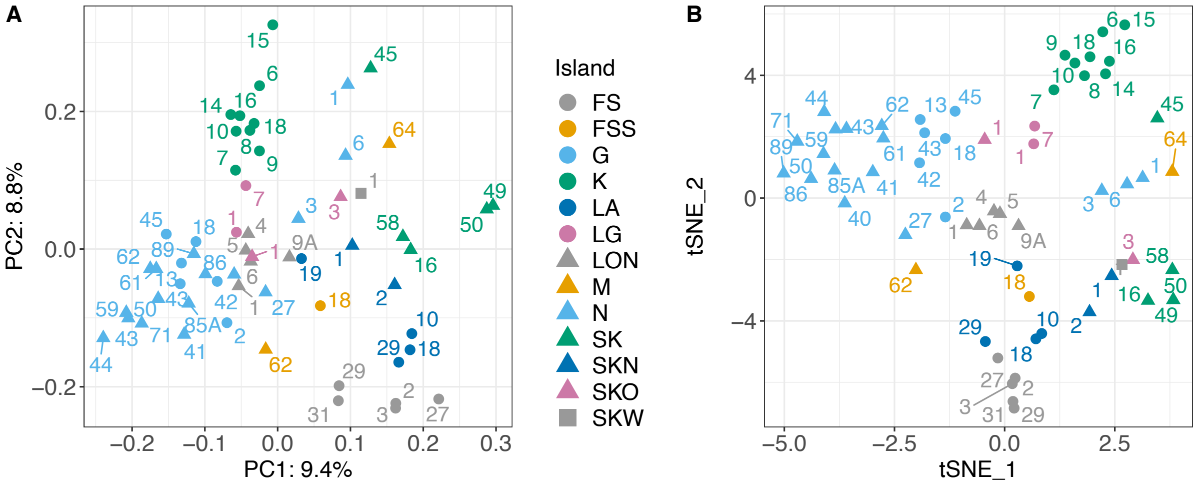

Figure S3: Association between (non-)synonymous genomic diversity and N_e. This is the same figure as Figure 3A but with numbers added to ponds. Observed π_N and π_S in the metapopulation. More diverse subpopulations have relatively fewer nonsynonymous polymorphisms. The color, symbol, and number scheme are the same as in Figure S1. The size of this symbol is indicative for the subpopulation's NN2. The black line indicates the one-to-one ratio.

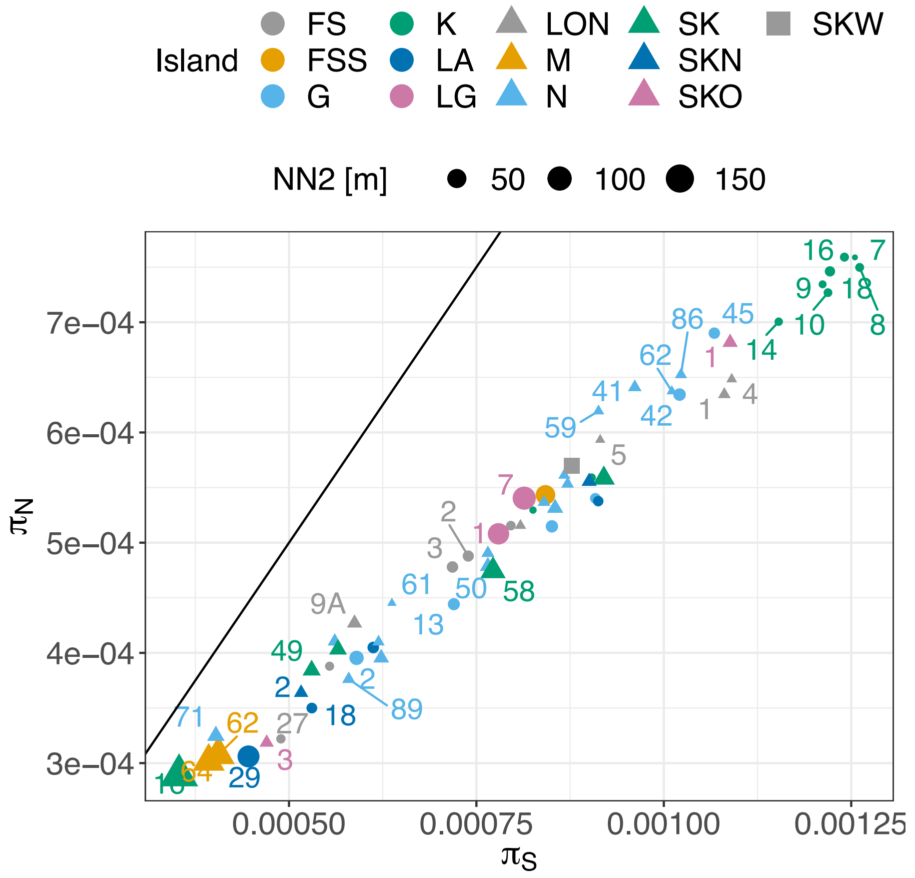

Figure S4: Association between nonsynonymous and synonymous genomic diversity in sexual populations of different sizes. This figure is similar to Figure 3B but shows simulations solely with sexual reproduction. Specifically, it shows simulated (non-)synonymous genetic diversity. The colored numbers show the simulated population sizes. H_S correlates well with N_e. Colors indicate the nonsynonymous distribution of fitness effects (DFE) following different gamma distributions (blue and red) or fixed to zero (green, total absence of selection; s = 0). Red shows the strongest selection. Error bars indicate the standard error around the mean of 1,000 runs. Colored lines are fits for the different nonsynonymous DFEs using linear regressions. The black line indicates the one-to-one ratio in both plots.

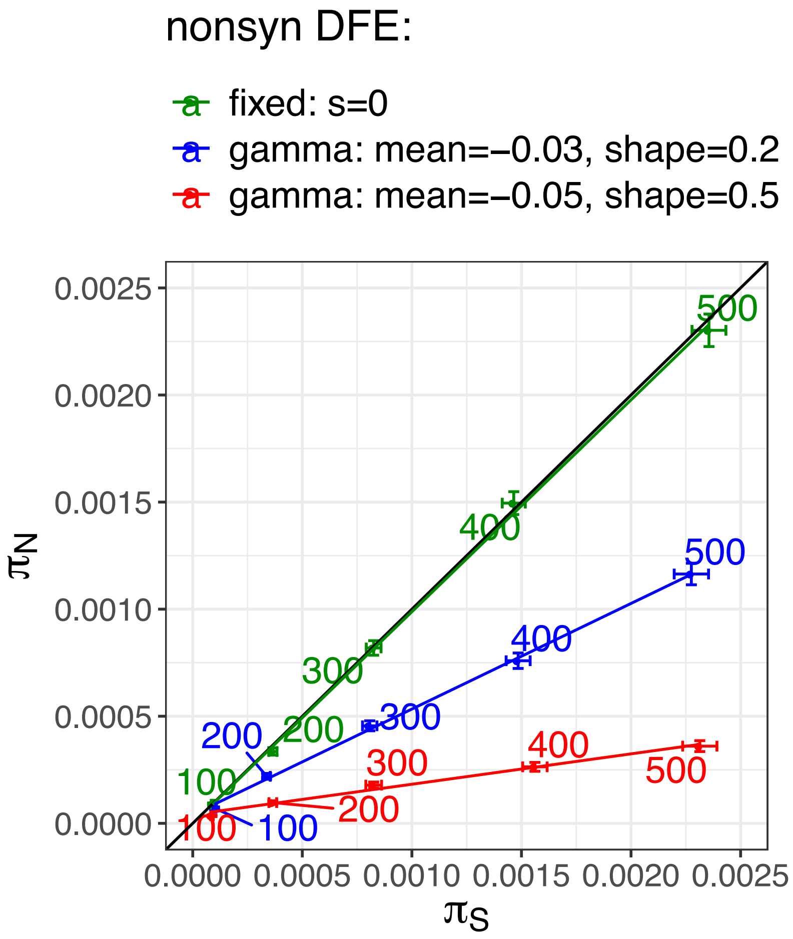

Figure S5: Association between nonadaptive nonsynonymous substitutions and π_S. Depicted is observed ω_{NA} of each subpopulation of the metapopulation. ω_{NA} is negatively associated with π_S. π_S can be used as an approximation for N_e. The red dot is the estimate for the single, large, stable population from Switzerland.

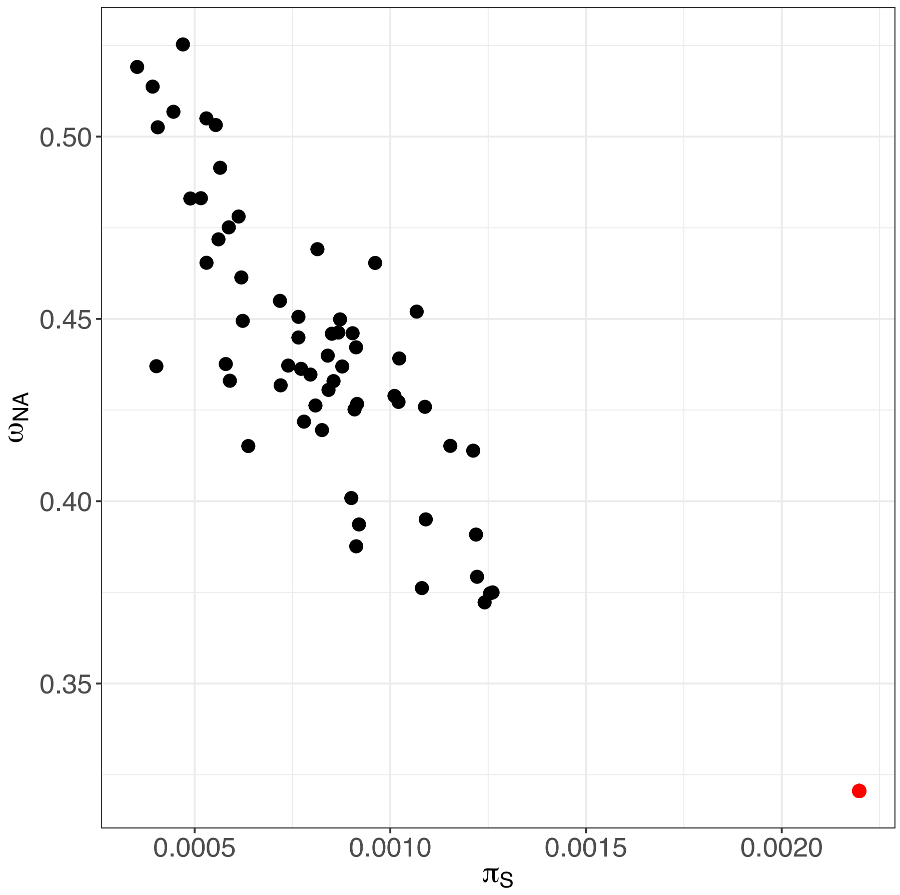

Figure S6: Simulated rate of nonadaptive nonsynonymous substitutions in differently sized populations. ω_{NA} is negatively associated with synonymous heterozygosity, π_S, and simulated population size. π_S can also be used as an approximation for N_e. Horizontal and vertical error bars indicate the standard error around the mean of 1,000 runs (simulations were made using SLiM).

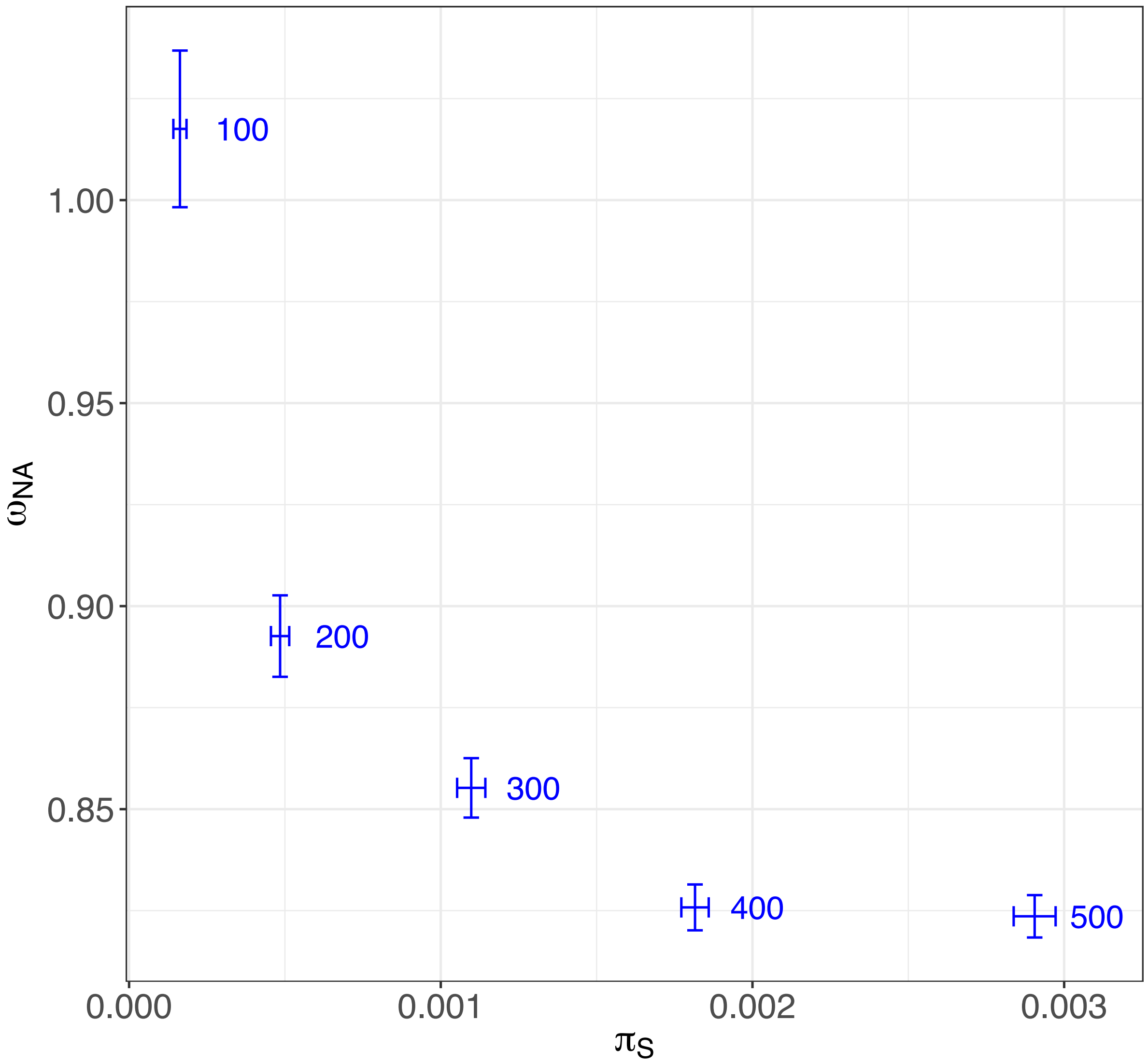

Figure S7: Genomic diversity explained by subpopulation age and isolation. This is the same figure as Figure 6 but with numbers added to ponds. Genomic diversity, π, is positively correlated with subpopulation age and negatively correlated with the isolation measure, NN2. The subpopulation age was added to one before log_{10}-transformation. The color, symbol, and number scheme are the same as in Figure S1. The size of this symbol is indicative for the subpopulation's NN2. The black line is a regression line based on a linear model with the confidence interval depicted as shading around the line.

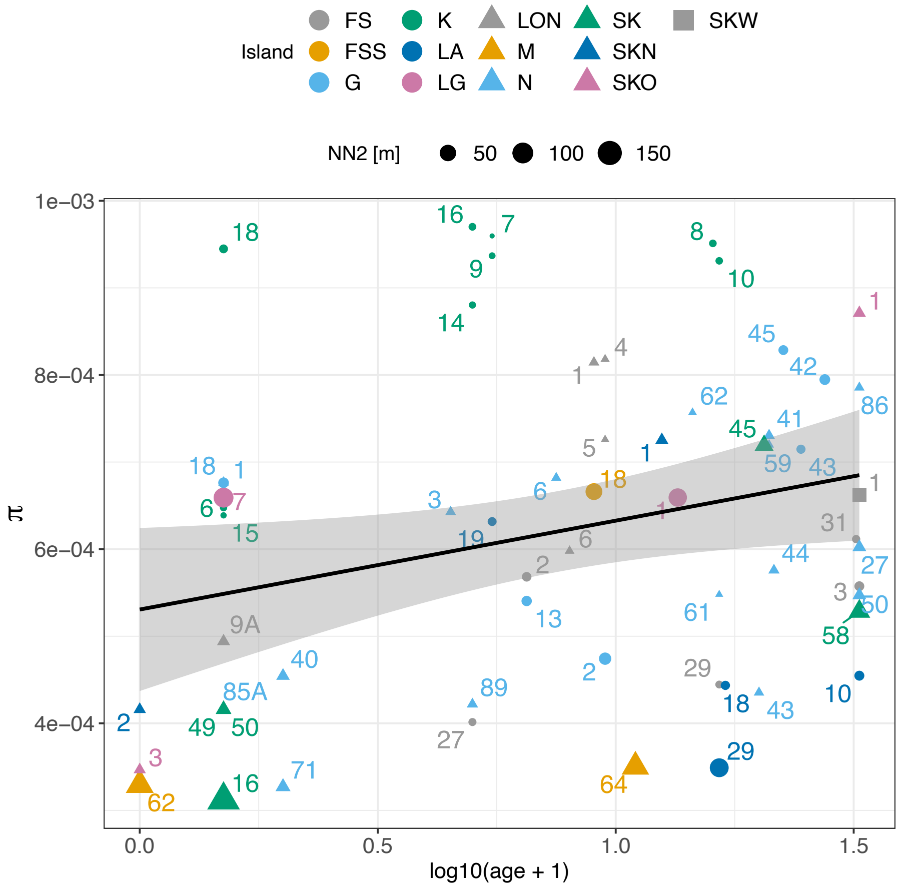

Figure S8: Isolation by distance (IBD). This is the same figure as Figure 7 but with numbers added to ponds. *F*-model-based F_{ST} positively correlates with the isolation measure NN2 [m]. Statistics derive from a Spearman correlation test. The color, symbol, and number scheme are the same as in Figure S1.

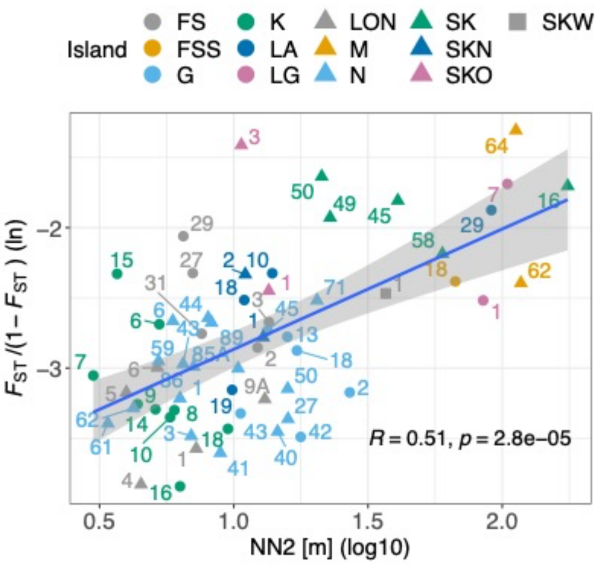

Figure S9: Isolation by distance (IBD). Pairwise population differentiation is positively correlated with geographic isolation measured as NN2. Each point represents the comparison between two subpopulations. Statistics derive from dbMEM analysis by RDA. The blue line is the regression line of a linear model with the 95 % confidence interval depicted as shading around the line.

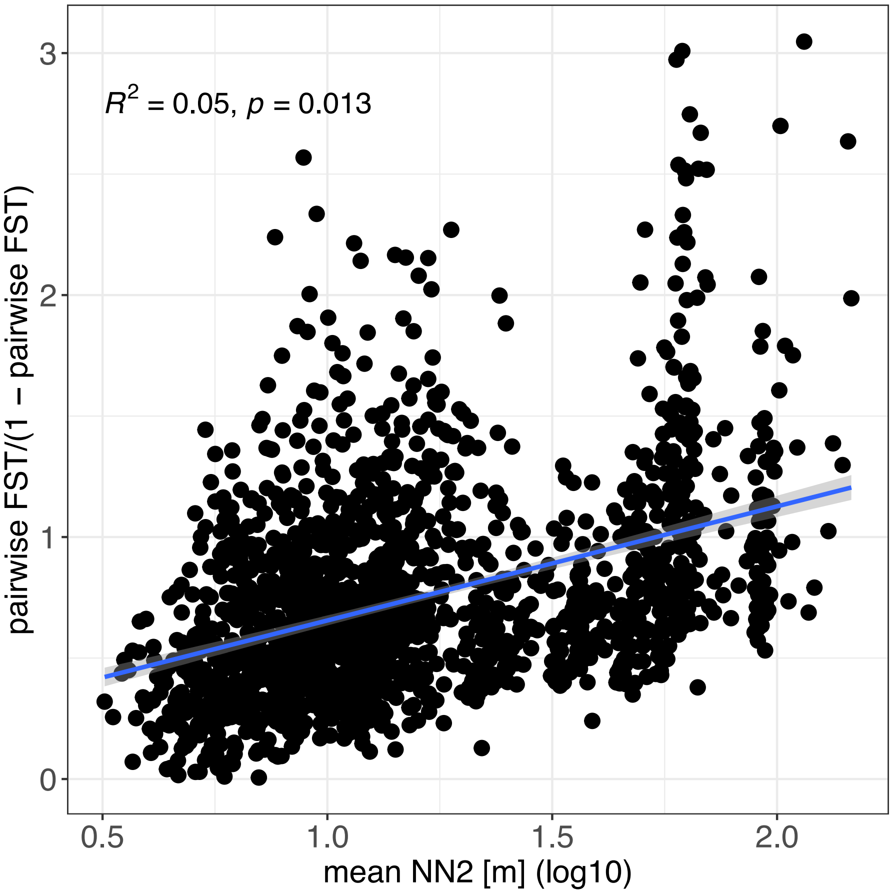

Figure S9: Association between nonsynonymous (π_N) and synonymous (π_S) in a single, relatively short contig. This Figure is similar to Figure 3A but shows the analysis results of a single, relatively short contig. It shows the same overall relationship between piN and piS but shows much more noise.

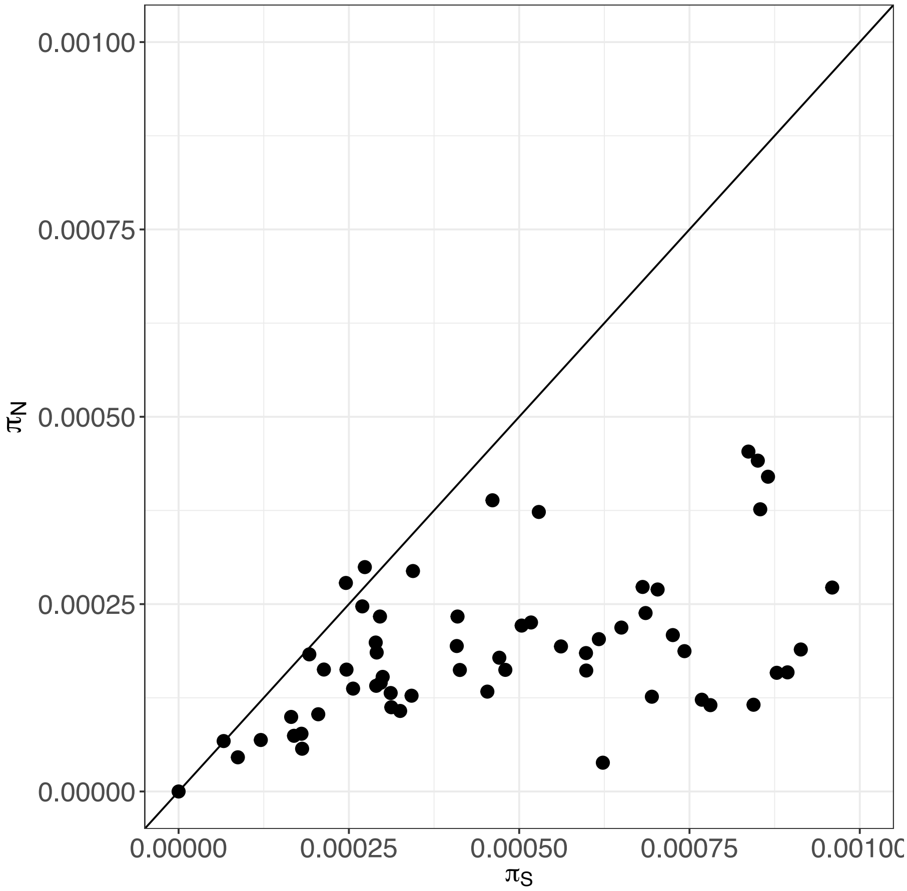

Table S1: Detailed sample information and mapping statistics. Each subpopulation sample (Pond ID) is accompanied by the number of pooled animals prior to DNA extraction (#animals), the percentage of sequencing reads that mapped to the *D. magna* reference genome (mapping_percent), the average whole-genome coverage (average_coverage), and the infection status with *H. tvaerminnensis* (infection_status).

Pond ID	#animals	mapping_percent	average_coverage	infection_status
FS-2	9	0.9886	84.383	0
FS-27	27	0.9399	22.6618	1
FS-29	17	0.9925	50.4757	0
FS-3	11	0.9649	67.5036	1
FS-31	31	0.918	53.9897	1
FSS-18	52	0.9886	76.6795	1
G-13	38	0.8331	19.9154	1
G-18	51	0.9872	66.0887	0
G-2	19	0.9721	62.5149	0
G-33	39	0.9799	0.321314	NA
G-42	51	0.9838	56.1439	1
G-43	50	0.9507	23.5622	1
G-45	44	0.9876	55.301	0
K-10	28	0.8706	45.5364	1
K-14	66	0.9432	48.357	1
K-15	67	0.8737	30.4633	1
K-16	54	0.8821	59.2523	1
K-18	61	0.8831	64.912	1
K-6	53	0.9642	21.4087	1
K-7	71	0.9766	104.367	1
K-8	53	0.8848	62.1974	1
K-9	58	0.9571	50.9035	1
LA-10	28	0.9823	21.1503	1
LA-18	30	0.9877	20.8245	1
LA-19	71	0.9845	21.7759	1
LA-29	59	0.9804	24.0561	0
LG-1	40	0.9873	64.8407	1
LG-7	49	0.9889	43.4276	0
LON-1	58	0.8055	37.0342	1
LON-4	55	0.7394	31.6327	1
LON-5	53	0.9572	48.7995	1
LON-6	51	0.9094	20.9864	1
LON-9A	14	0.955	25.4082	1
M-62	50	0.948	24.7763	1
M-64	42	0.9871	47.523	0

N-1	11	0.9729	46.767	1
N-27	49	0.9625	20.2174	1
N-3	56	0.931	18.6522	1
N-40	46	0.8756	18.6395	1
N-41	49	0.9925	61.2961	0
N-43	53	0.9034	16.1057	1
N-44	49	0.938	23.3142	1
N-50	54	0.9432	21.2262	1
N-59	36	0.8319	54.2554	1
N-6	53	0.9571	36.5148	1
N-61	37	0.7418	17.5543	1
N-62	57	0.9247	36.0808	1
N-71	54	0.7249	15.3076	1
N-85A	54	0.8932	18.5148	1
N-86	49	0.979	51.3107	1
N-89	50	0.8405	30.9543	1
SK-1	61	0.9458	0.230235	NA
SK-16	60	0.9866	20.1261	0
SK-45	37	0.8716	43.4562	1
SK-49	50	0.9935	27.6059	0
SK-50	48	0.9888	47.9354	0
SK-58	48	0.9683	19.4414	1
SKN-1	49	0.9006	52.9265	1
SKN-2	61	0.9744	21.7282	1
SKO-1	45	0.9706	57.1013	1
SKO-3	51	0.9883	37.4972	0
SKW-1	50	0.9862	23.9365	1

Table S2: Type two analysis of variance between genomic diversity, π, and explanatory variables, including the island of origin. After including the island of origin, the association to the measure of isolation became weaker and insignificant, since isolated subpopulations are mostly from the same, isolated islands ($F(12,47) = 13.57$, $p < 0.001$, $R^2 = 0.776$). One R^2 value is given for the complete model. Significant associations are in bold.

Explanatory variable	df	Statistics	*p*	R^2
Population age	42	t = 2.712	**0.0004**	0.673
PC1 (marineness)	42	t = -0.822	0.642	
PC2 (pond geometry)	42	t = 0.259	0.874	
NN2 (isolation)	42	t = -1.538	0.060	
Island	12/42	F = 5.0021	$\mathbf{4.4 \times 10^{-5}}$	
Infection status	1/42	F = 0.1464	0.704	
